# A New Method for Detecting Associations with Rare Copy-Number Variants

**DOI:** 10.1371/journal.pgen.1005403

**Published:** 2015-10-02

**Authors:** Jung-Ying Tzeng, Patrik K. E. Magnusson, Patrick F. Sullivan, The Swedish Schizophrenia Consortium, Jin P. Szatkiewicz

**Affiliations:** 1 Department of Statistics and Bioinformatics Research Center, North Carolina State University, Raleigh, North Carolina, United States of America; 2 Department of Statistics, National Cheng-Kung University, Tainan, Taiwan; 3 Department of Genetics, University of North Carolina at Chapel Hill, Chapel Hill, North Carolina, United States of America; 4 Department of Medical Epidemiology and Biostatistics, Karolinska Institutet, Stockholm, Sweden; Children's Hospital of Philadelphia, UNITED STATES

## Abstract

Copy number variants (CNVs) play an important role in the etiology of many diseases such as cancers and psychiatric disorders. Due to a modest marginal effect size or the rarity of the CNVs, collapsing rare CNVs together and collectively evaluating their effect serves as a key approach to evaluating the collective effect of rare CNVs on disease risk. While a plethora of powerful collapsing methods are available for sequence variants (e.g., SNPs) in association analysis, these methods cannot be directly applied to rare CNVs due to the CNV-specific challenges, i.e., the multi-faceted nature of CNV polymorphisms (e.g., CNVs vary in size, type, dosage, and details of gene disruption), and etiological heterogeneity (e.g., heterogeneous effects of duplications and deletions that occur within a locus or in different loci). Existing CNV collapsing analysis methods (a.k.a. the burden test) tend to have suboptimal performance due to the fact that these methods often ignore heterogeneity and evaluate only the marginal effects of a CNV feature. We introduce CCRET, a random effects test for collapsing rare CNVs when searching for disease associations. CCRET is applicable to variants measured on a multi-categorical scale, collectively modeling the effects of multiple CNV features, and is robust to etiological heterogeneity. Multiple confounders can be simultaneously corrected. To evaluate the performance of CCRET, we conducted extensive simulations and analyzed large-scale schizophrenia datasets. We show that CCRET has powerful and robust performance under multiple types of etiological heterogeneity, and has performance comparable to or better than existing methods when there is no heterogeneity.

## Author Summary

Copy number variants (CNVs) are the gain or loss of DNA segments in the genome that can vary in dosage, length and details of gene disruptions. Rare CNVs have been shown to be associated with neuropsychiatric disorders both collectively and at specific loci. To evaluate the collective effects of rare CNVs on disease risk, sophisticated association methods are needed to pool information across CNV loci while handling CNV-specific properties; however, such methods are under-developed. To address these challenges, we have developed a new collapsing method for rare CNVs named CCRET. CCRET is a random effects approach applicable to variants measured on a multi-categorical scale, collectively modeling the effects of multiple CNV features, and is robust to etiological heterogeneity. Multiple confounders can be simultaneously corrected. To evaluate the performance of CCRET, we conducted extensive simulation and analyzed large-scale schizophrenia datasets. We demonstrate the robustness, validity and utility of CCRET under a variety of scenarios.

## Introduction

Copy-number variants (CNVs), such as deletions and duplications, are changes in the number of DNA copies (in comparison to the reference) and are a major source of genetic variation in the human genome [1–3]. While a sequence variant (e.g., a SNP) affects a single nucleotide, a CNV affects a region > 1 Kb. CNV may alter the “dosage” of one or more genes or regulatory regions in the deleted or duplicated region, which can consequently exert a profound effect on the risk for human disease. Genomic evaluation of CNVs has established a role for rare (<1%) CNVs in the etiology of psychiatric disorders, such as schizophrenia, bipolar, and autism [4–6]. Eight rare CNVs of strong effects are now established risk factors for psychiatric disorders (e.g., 16p11.2, 22q11.2, genotypic relative risk 4–20) [4, 5]. However, the bulk of CNVs’ contribution to disease risk remains unknown due to a modest effect size or the rarity of the CNV. As psychiatric disorders are polygenic, collapsing methods [7], which collapse multiple variants into a group and evaluate their collective effect on disease risk, serve as key approaches to the analysis of rare CNVs [4, 5]. By accumulating information across multiple rare variants (e.g., counting the number of mutations for each individual), collapsing methods can have an enhanced power to detect genetic variants that are hard to detect individually but collectively show a significant impact. For example, multiple studies have confirmed a greater genomewide burden of rare CNVs in schizophrenia cases compared with controls [8–12]. Enrichment analyses of genes impacted by rare CNVs implicated several biological pathways important to schizophrenia, including those previously associated with schizophrenia through common variation and exome sequencing (e.g., calcium channel signaling and binding partners of the fragile X mental retardation protein) [12].

For rare sequence variants such as SNPs, a plethora of powerful methods are available to perform collapsing analysis. Depending on the approaches used to model genetic effects and the procedures used to collapse the information across loci, these methods can be classified into two major categories: fixed effects methods (e.g., CMC [13], VT [14]) and random effects methods (e.g., C-alpha [15], SKAT [16], SimReg [17, 18]). A detailed review can be found in Pongpanich et al. [19] and Lee et al. [20]. Briefly, fixed effects methods collapse information at genotype level and assess the mean level of the genetic effects via fixed effects modeling; it is the optimal approach if the effects of different loci are additive and of a similar size. In contrast, random effects methods collapse information at similarity level and assess the variance level of the genetic effects via random effects modeling. Random effects methods are more powerful than fixed effects methods when the variants have different effects (e.g., mixture of positive, negative and neutral effects).

However, SNP collapsing methods cannot be straightforwardly applied to CNVs due to several CNV-specific challenges. First, copy number is measured on a multi-categorical scale (e.g., duplication, normal copy and deletion) while SNP collapsing methods assume binary events (e.g., mutation vs. no mutation). Second, CNV can vary in dosage (i.e., the copy number of a CNV), length (i.e., the segment size of a CNV) and details of gene disruptions (i.e., the number of genes that a CNV intersects), and each of these “features” affects CNVs’ impact on disease risk. For example, in schizophrenia, deletions were enriched in cases to a greater extent than duplications, and the largest CNVs (> 500 kb) were enriched in cases to a greater extent than other size categories [8–12]. Girirajan et al. [21] found that the total duplication length is significantly elevated in autism cases compared with controls. On the other hand, SNP collapsing methods target only one feature (i.e., mutation burden). Third, etiological heterogeneity is often observed in CNVs. While SNPs only exhibit between-locus heterogeneity, the etiological heterogeneity of CNVs can occur both between loci (e.g., CNVs across different loci have different effects on disease risk) and within a locus (i.e. different dosages of CNV alleles within the same locus have different effects). (Please see “Input data format” in the Method Section for detailed definitions of “locus”.) For example, the 22q11.2 deletion is a known risk factor for schizophrenia [4, 5, 22–24], whereas the reciprocal 22q11.2 duplication is potentially a protective factor [25]. Microduplications of gene *VIPR2* increase the risk for schizophrenia, where both tandem duplication (copy number 3) and triplication (copy number 4) were observed with triplication potentially conveying higher risk than duplication [26]. The etiological heterogeneity may occur more frequently as CNV detection technologies continue to improve, allowing the accurate detection of small CNVs. Naïve collapsing of a mixture of neutral, risk, and protective variants between loci or within a locus can cancel signals and lead to power loss. Random effects SNP-collapsing methods have the potential to address between-locus etiological heterogeneity in CNV analyses; however, because these methods record the genetic information using the number of mutant events, they cannot deal with the within-locus etiological heterogeneity observed in multi-categorical scale CNVs.

Collapsing methods based on fixed effects approaches have been developed for rare CNVs [9, 27]. Specifically, burden-style tests [9] examine CNV events to evaluate whether an increased rate or the size of the CNVs increases disease risk. Rare CNVs are typically aggregated based on a certain event of interest and then summarized by the event counts, such as the number of deletions (copy number <2), the number of duplications (copy number >2), the number of CNVs (copy number≠2), or the number of genes intersected by the CNVs. The association is evaluated by comparing the event rates between cases and controls. However, this approach falls short in three aspects. First, the researcher must pre-select a CNV event (e.g., deletion or duplication or both) and summarize the data using the appropriate event counts. Second, it ignores etiological heterogeneity. Finally, it only evaluates the marginal effects of a CNV feature, which may or may not be a valid approach depending on the collapsing unit. For example, while the gene-count burden test is valid for a genomewide collapsing, it can result in spurious association if based on a gene set [27]. To address the last issue, enrichment-style tests [27] have been proposed to assess the conditional effect of a CNV feature; such a test compares the counts of genes within a pre-specified gene set intersected by CNVs in cases with the counts in controls while controlling for case-control differences in genomewide CNV rate and size. Although it uses a joint modeling to avoid spurious association, this method does not address the issue of etiological heterogeneity and still needs to summarize CNV data into counts based on the feature of interest.

We have developed a new collapsing method for the analysis of rare CNVs that is applicable to variants measured on a multi-categorical scale, collectively modeling the effects of multiple CNV features, and is robust to etiological heterogeneity. Our method is called CCRET (CNV Collapsing Random Effects Test, pronounced as “se-cret”). Specifically, we use random effects to model the CNV effect of interest, which, for SNP collapsing analysis, has been shown to be more robust than a fixed effects approach when the complexity of genetic architecture increases [19, 20]. When aggregating information through CNV similarity, we define “locus” units for each CNV feature to retain the “locus”-specific effect during collapsing, and use factorized design vectors for multi-categorical CNV features to quantify similarity without dichotomizing the data as is done in the fixed effects methods. CCRET can simultaneously correct for covariates such as batch effects and population substructures. CCRET can be viewed as an extension of the SNP-set random effects methods (e.g., C-alpha [15], SKAT [16], SimReg [17, 18]) applicable to CNV data. To evaluate the performance of CCRET, we conducted extensive simulations under various scenarios and analyzed large-scale CNV data from the Swedish schizophrenia study. Our results suggest that, compared with the existing (fixed effects) CNV collapsing methods implemented in PLINK [9, 27, 28], our random effects approach has a stable, powerful and robust performance under multiple types of etiological heterogeneity, and has a comparable or better performance when there is no heterogeneity.

## Results

### Overview of CCRET (CNV Collapsing Random Effects Test)

**Fig 1:**
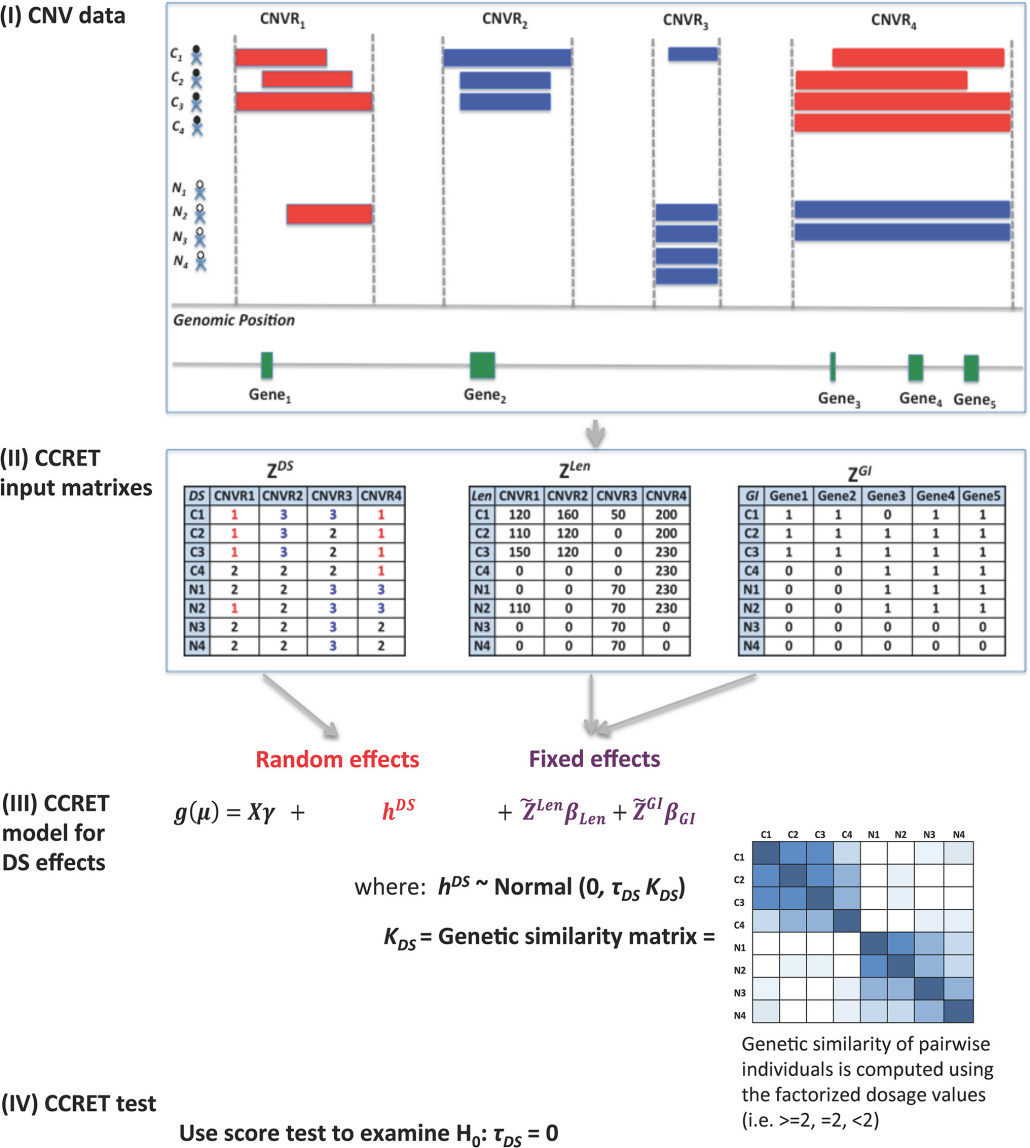
An overview of the CCRET method. The dosage effects model was used as an example. C_1-4_: cases, N_1-4_: controls, CNVR: copy number variation region, red rectangle: deletion, blue rectangle: duplication, green rectangle: gene. DS: dosage, Len: length, GI: gene intersection.

Fig 1 provides an overview of the CCRET method using the dosage effects model as an example. CCRET aims to detect any association of the aggregated CNV effect with disease risk and has the following key features. First, CCRET converts the source CNV data to three input matrixes in order to store the different features of CNVs, i.e., dosage (“DS”), length (“Len”), and gene intersection (“GI”). For “DS” and “Len” matrixes, we use CNV regions as the “locus” unit. For “GI” matrix, we use genes as the “locus” units. Second, CCRET models the covariates and background CNV features using fixed effects as did in Raychaudhuri et al [27], and models the CNV feature of interest using random effects in order to retain the locus-specific details and to account for both between-locus and within-locus etiological heterogeneity. Third, CCRET quantifies the genetic similarity between any two individuals based on the CNV feature of interest, which is then used to depict the covariance among the CNV effects of different individuals (i.e, the more similar the genetic feature between two individuals is, the more correlated their CNV effects would be). When calculating genetic similarity, we factorize the multi-categorical allele values recorded in the input matrices. Consequently, alleles with opposite effects within a locus are not lumped together when computing similarity, which makes CCRET robust against within-locus heterogeneity. In contrast, SNP-collapsing random effects methods (e.g., C-alpha [15], SKAT [16], SimReg [17, 18]) do not address within-locus heterogeneity. Finally, under the mixed effects model framework, the aggregate CNV effect can be evaluated by examining the significance of the variance component. In contrast, fixed effects methods test the aggregated CNV effect by examining if the common (e.g., averaged) effect is equal to zero.

**Fig 2:**
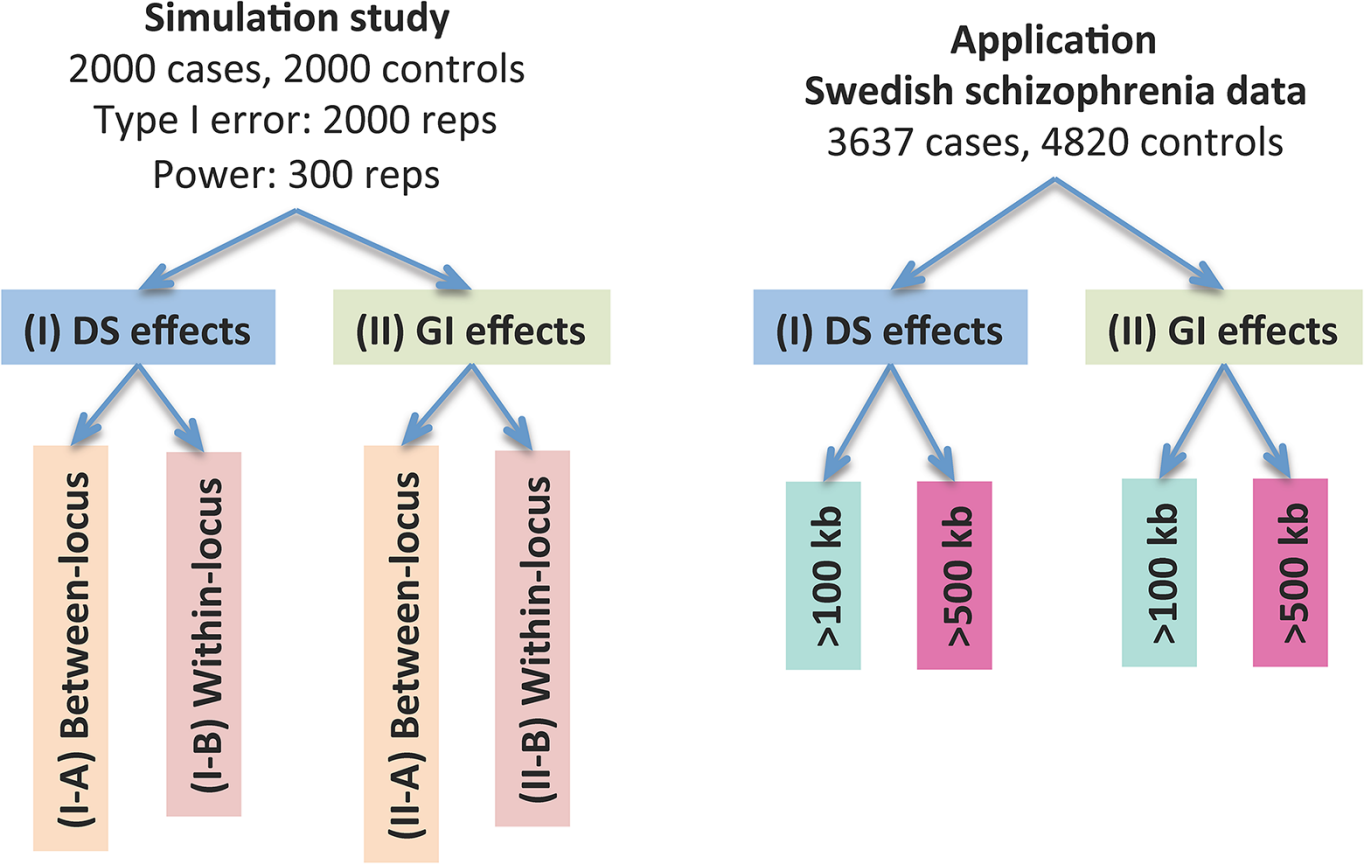
Performance evaluation of the CCRET method. “Between-locus”: between-locus heterogeneity; “Within-locus”: within-locus heterogeneity.

Fig 2 provides an overview of the evaluative analyses carried out in this work. We conducted two sets of simulations under a variety of scenarios and conducted real data analysis using large-scale schizophrenia datasets. We evaluated the performance of CCRET in comparison to the fixed effects CNV-collapsing methods implemented in PLINK [9, 27, 28].

### Simulation studies for performance evaluations

We conducted two sets of simulations to evaluate the performance of our method under a variety of scenarios: (I) causal dosage effects and (II) causal gene intersection (GI) effects. In each simulation, the genotypes of rare CNVs were taken from the TwinGene study [29, 30], which used a cross-sectional sampling design and selected 6,611 unrelated subjects born between 1911 and 1958 from the Swedish Twin Registry [31–33] (STR) for genomic characterization. These samples included one member per monozygotic twin pair and a randomly selected member per dizygotic twin pair. DNA was extracted from peripheral venous blood for all subjects. The samples were genotyped on the Illumina OmniExpress beadchip for 72,881 SNP markers using standard protocol; CNV calling was performed using PennCNV [34] (version June 2011) with recommended model parameters. We randomly selected 2,000 subjects from the TwinGene study and formed 1,757 CNV regions (CNVRs) genome-wide with frequency <1%. The detailed data processing procedure is described in ***S1 Text***. In each simulation setting, we used case-control sampling to collect 2,000 cases and 2,000 controls, evaluated the type I error rates based on 2,000 replications and evaluated the power based on 300 replications.

#### Simulation I: evaluating dosage effects

We considered causal dosage effects with two types of etiological heterogeneity between deletions and duplications: (A) between-locus heterogeneity, and (B) within-locus heterogeneity. We simulated the binary phenotype of individual *i* from the Bernoulli distribution with *π*_*i*_ as the probability of being a case and $logit(\pi_{i})=-2+\sum_{m}\beta_{m}^{DS.Dup}\times z_{im}^{DS.Dup}+\sum_{m}\beta_{m}^{DS.Del}\times z_{im}^{DS.Del}$, where $z_{im}^{DS.Dup}=1$ if individual *i* at causal locus *m* has a duplication and 0 otherwise; $z_{im}^{DS.Del}$ is defined in a similar fashion for deletion events; $\beta_{m}^{DS.Dup}$ and $\beta_{m}^{DS.Del}$ are the log of the odds ratios (ORs) of causal locus *m* for duplications and deletions, respectively; $\beta_{m}^{DS.Dup}$ and $\beta_{m}^{DS.Del}$ shared the same absolute values for all causal loci but were positive if associated with increased disease risk and negative if protective effects. We compared CCRET to the collapsing methods implemented in PLINK. Specifically, PLINK command “—cnv-indiv-perm” was applied to duplications only (referred to as PLINK.dup), deletions only (referred to as PLINK.del), and deletions and duplications combined (referred to as PLINK.all). The PLINK p-values were computed using 10,000 permutations.

#### Simulation I-A: between-locus heterogeneity of the dosage simulation

Among the 1,757 CNVRs, there are 766 loci with duplication genotypes only (dosage 3 or 4, “DupOnly” hereafter), 840 loci with deletion genotypes only (dosage 0 or 1, “DelOnly” hereafter), and 151 loci with both deletion and duplication genotypes (dosage 0,1,3,4, “DupDel” hereafter). We considered 600 causal loci where 300 loci were randomly selected from the 766 DupOnly loci and 300 loci were randomly from the 840 DelOnly loci. We considered 6 heterogeneity models with different proportions of risk-associated ($\beta_{m}^{DS.•}>0$) or protective ($\beta_{m}^{DS.•}<0$) effects among causal loci. The first three served as the baseline models which favor the PLINK burden tests, i.e., (1) all DupOnly risk-associated and all DelOnly protective (i.e., *β* ^*DS*.*Dup*^ +/− = 100/0 and *β* ^*DS*.*Del*^ +/− = 0/100); (2) all DupOnly protective and all DelOnly risk-associated (i.e., *β* ^*DS*.*Dup*^ +/− = 0/100 and *β* ^*DS*.*Del*^ +/− = 100/0); (3) all causal loci risk-associated (i.e., no heterogeneity) with *β* ^*DS*.*Dup*^ +/− = 100/0 and *β* ^*DS*.*Del*^ +/− = 100/0. The remaining three models consider different levels of heterogeneity, i.e., (4) *β* ^*DS*.*Dup*^ +/− = 70/30 and *β* ^*DS*.*Del*^ +/− = 30/70; (5) *β* ^*DS*.*Dup*^ +/− = 30/70 and *β* ^*DS*.*Del*^ +/− = 70/30, and (6) *β* ^*DS*.*Dup*^ +/− = 50/50 and *β* ^*DS*.*Del*^ +/− = 50/50. For each scenario, we assumed a constant OR for casual loci and considered OR ranging between 1 and 7, chosen based on the empirical evidence of pathogenic CNVs in psychiatric disorders [4, 5].

**Fig 3:**
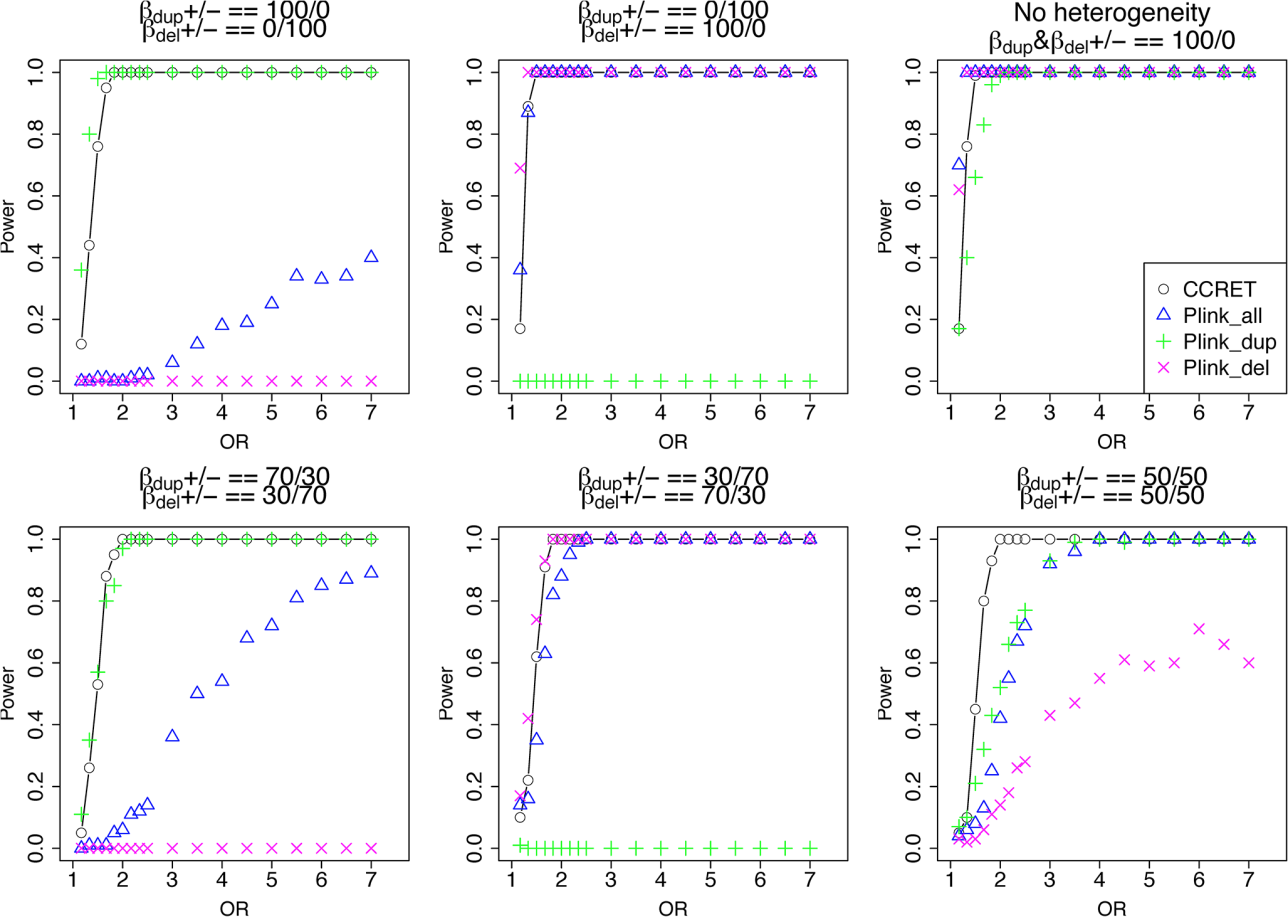
Power comparison between CCRET and PLINK 1-sided tests for simulation I-A: between-locus heterogeneity of the dosage simulation. As detailed in section “Simulation Design”, we considered 6 heterogeneity models with different proportions of risk-associated ($\beta_{m}^{DS.•}>0$) or protective ($\beta_{m}^{DS.•}<0$) effects among causal loci. Black **O** line: CCRET; Blue **Δ**: PLINK 1-sided test analyzing deletions and duplications combined; Green **+**: PLINK 1-sided test analyzing only duplications; Magenta **x**: PLINK 1-sided test analyzing only deletions.

The type I error rates were around the nominal level for all methods under between-locus, although the results of CCRET are slightly conservative (Table 1). For power analyses, we first compared CCRET to PLINK 1-sided tests, which assess whether the event rate is higher in cases than in controls (Fig 3). In models (1) to (3) (Fig 3 upper panel, where duplications (deletions) have the same effects and PLINK tests would be the most powerful), CCRET provided comparable power to the best PLINK methods while the best PLINK method varied. Specifically, in (1) where all duplications were risk-associated and all deletions were protective, PLINK.dup was the best method as expected; similarly, in (2), PLINK.del was the best method as expected; and in (3), PLINK.all was the best method, because PLINK.all used all available information whereas PLINK.dup or PLINK.del only used a subset of the total events. In Models (4) to (6), (Fig 3 lower panel, where different combinations existed of risk-associated and protective effects in *β* ^*Dup*^ and *β* ^*Del*^), CCRET consistently yielded the best power or yielded power comparable to the best PLINK method. From Fig 3, we also see that the performance of PLINK 1-sided tests was highly dependent on the underlying effect mechanisms. Specifically, the best PLINK 1-sided tests were those focused on the CNV events with risk-associated effect, and those PLINK 1-sided tests that focused on the events with protective effects had no power. In the presence of etiological heterogeneity (i.e., Models (1), (2), and (4) through (6)), the performance of PLINK.all was hard to predict; roughly speaking, it tended to be in and between PLINK.dup and PLINK.del, and the power somewhat depended on the relative proportion of the causal risk-associated and causal protective CNVRs.

**Table 1:** Type I error rates for evaluating dosage effects (nominal alpha = 0.05).

Model	CCRET	PLINK.all	PLINK.dup	PLINK.del
(A) Between-locus heterogeneity	0.035	0.046*	0.057	0.041
		(0.047)**	(0.053)	(0.055)
(B) Within-locus heterogeneity	0.041	0.051	0.057	0.043

*: Type I error rates based on PLINK 2-sided tests

**: Type I error rates in parentheses are based on PLINK 1-sided tests.

**Fig 4:**
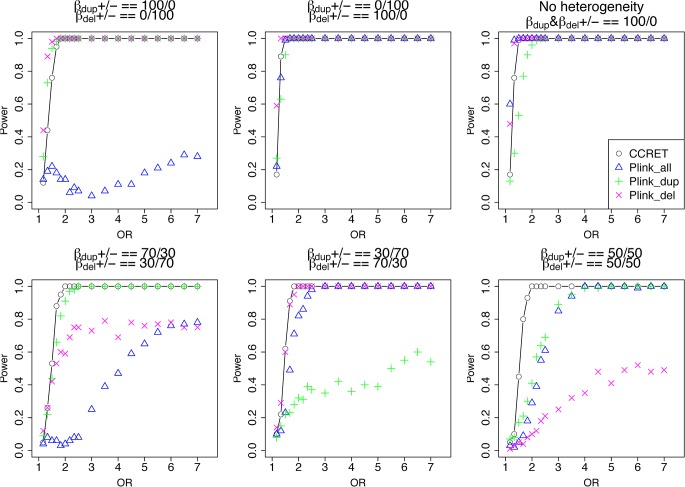
Power comparison between CCRET and PLINK 2-sided tests for simulation I-A: between-locus heterogeneity of the dosage simulation, under 6 heterogeneity models as in Fig 3. Black **O** line: CCRET; Blue **Δ**: PLINK 2-sided test analyzing deletions and duplications combined; Green **+**: PLINK 2-sided test analyzing only duplications; Magenta **x**: PLINK 2-sided test analyzing only deletions.

In Fig 4, we compare CCRET to PLINK 2-sided tests. For PLINK 2-sided tests, we observed a pattern of the relative performance of PLINK and CCRET similar to that seen in Fig 3. The only exception was that PLINK.dup and PLINK.del had good power under heterogeneity models (1) and (2). In sum, PLINK 2-sided tests are more robust than PLINK 1-sided tests when the underlying effect patterns are unknown; and therefore we present the comparisons of CCRET and PLINK 2-sided tests for the remaining simulation studies.

#### Simulation I-B: within-locus heterogeneity of the dosage simulation

We selected 38 loci out of the 151 DupDel loci to be causal and the selected causal loci tended to have a similar number of duplications and deletions. We considered 5 heterogeneity models, where deletions and duplications had different combinations of risk-associated (R), protective (P) or neutral (N) effects on the phenotypes. Specifically, the five effect combinations considered were (Dup, Del) = (R,N), (N,R), (P,R), (R,P) and (R,R) (i.e., no heterogeneity). For example, “(Dup, Del) = (P,R)” indicates that, within each of the 38 loci, duplications had protective effects, whereas deletions had risk-associated effects. For each model, we set a constant OR for the casual loci and considered OR ranging between 1 and 20, chosen based on empirical evidences [4, 5].

**Fig 5:**
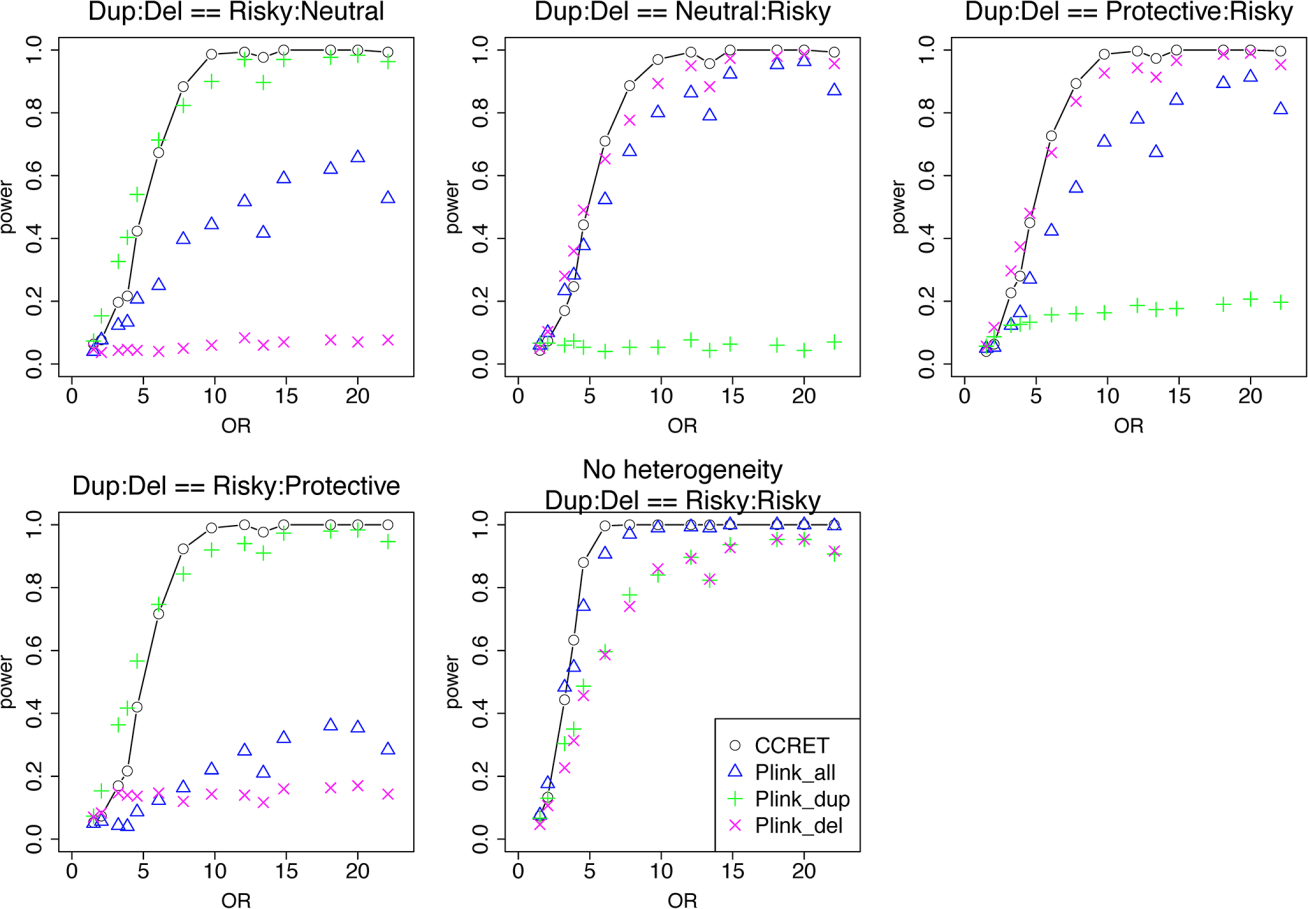
Power comparison between CCRET and PLINK 2-sided tests for simulation I-B: within-locus heterogeneity of the dosage simulation, under 5 heterogeneity models as detailed in section “Simulation Design”. Black **O** line: CCRET; Blue **Δ**: PLINK 2-sided test analyzing deletions and duplications combined; Green **+**: PLINK 2-sided test analyzing only duplications; Magenta **x**: PLINK 2-sided test analyzing only deletions.

Table 1 shows that the type I error rates were around the nominal level for all methods. The power results (Fig 5) show that CCRET had power comparable to or better than the best PLINK test across all heterogeneity models considered. Again, the best PLINK test varied across heterogeneity models, but overall it focused on the CNV allele with risk-associated effects. The PLINK tests that focused on the protective (neutral) alleles had low (no) power. PLINK.all had power similar to and between PLINK.dup and PLINK.del, except in the case where within-locus heterogeneity did not exist, i.e., (R,R).

#### Simulation II: evaluating GI effects

In Simulation II, we considered causal GI with heterogeneous effects between a duplication GI and deletion GI. In the TwinGene data, we focused on 668 protein-coding genes in the neuronal postsynaptic density (PSD) because previous researchers have reported enrichment of rare CNVs in the PSD genes in schizophrenia cases [12, 35]. CNVs intersected 69 of the 668 genes, where 35 genes were intersected by duplications only (“DupOnly genes” hereafter), 26 genes were intersected by deletions only (“DelOnly genes” hereafter), and 8 genes were intersected by both deletions and duplications (“DupDel genes” hereafter). Similar to the first simulation study, we considered (A) between-gene heterogeneity and (B) within-gene heterogeneity. We simulated the binary phenotype of individual *i* from the Bernoulli distribution with *π*_*i*_ as the probability of being a case and $logit(\pi_{i})=-2.5+\sum_{m}\beta_{m}^{GI.Dup}\times z_{im}^{GI.Dup}+\sum_{m}\beta_{m}^{GI.Del}\times z_{im}^{GI.Del}+\beta^{CNV}\times z_{CNV,i}+\beta^{Len}\times z_{Len,i}$, where $z_{im}^{GI.Dup}=1$ if causal gene *m* is intersected by a duplication and 0 otherwise; $z_{im}^{GI.Del}$ is defined similarly for deletion; *z*_*CNV*,*i*_ is the total number of CNV events; and, *z*_*Len*,*i*_ is the mean size of the CNVs measured in kb. The regression coefficients $\beta_{m}^{GI.Dup}$ and $\beta_{m}^{GI.Del}$ are the log(OR)’s of causal gene *m* for a duplication intersection and a deletion intersection, respectively; $\beta_{m}^{GI.Dup}$ and $\beta_{m}^{GI.Del}$ shared the same absolute values for all causal loci but were positive if risk-associated effects and negative if protective effects; *β* ^*CNV*^ and *β* ^*Len*^ were set to be log(1.5). We compared CCRET with the PLINK enrichment test as described in Raychaudhuri et al. [27]. We implemented the procedure in R and reported the 2-sided asymptotic *p*-values as done in the default option of PLINK “—cnv-enrichment-test”. We performed the enrichment analysis for deletions and duplications combined (referred to as PLINK.enrich), deletions only (PLINK.enrich.del), and duplications only (PLINK.enrich.dup).

#### Simulation II-A: between-locus heterogeneity of the GI simulation

We randomly selected 26 genes from the DupOnly genes and 26 genes from the DelOnly genes, and used the 52 genes as causal loci. We considered 6 heterogeneity models similar to the models considered in the dosage simulation: (1) DupOnly risk-associated and DelOnly protective (i.e., *β* ^*GI*.*Dup*^ +/− = 100/0 and *β* ^*GI*.*Del*^ +/− = 0/100); (2) DupOnly protective and DelOnly risk-associated (i.e., *β* ^*GI*.*Dup*^ +/− = 0/100 and *β* ^*GI*.*Del*^ +/− = 100/0); (3) all causal loci risk-associated (i.e., no heterogeneity with *β* ^*GI*.*Dup*^ +/− = 100/0 and *β* ^*GI*.*Del*^ +/− = 100/0); (4) *β* ^*GI*.*Dup*^ +/− = 70/30 and *β* ^*GI*.*Del*^ +/− = 30/70; (5) *β* ^*GI*.*Dup*^ +/− = 30/70 and *β* ^*GI*.*Del*^ +/− = 70/30; and (6) *β* ^*GI*.*Dup*^ +/− = 50/50 and *β* ^*GI*.*Del*^ +/− = 50/50. We set a constant effect size for all casual loci and considered the OR ranging between 1 and 20, chosen based on empirical evidences [4, 5].

**Fig 6:**
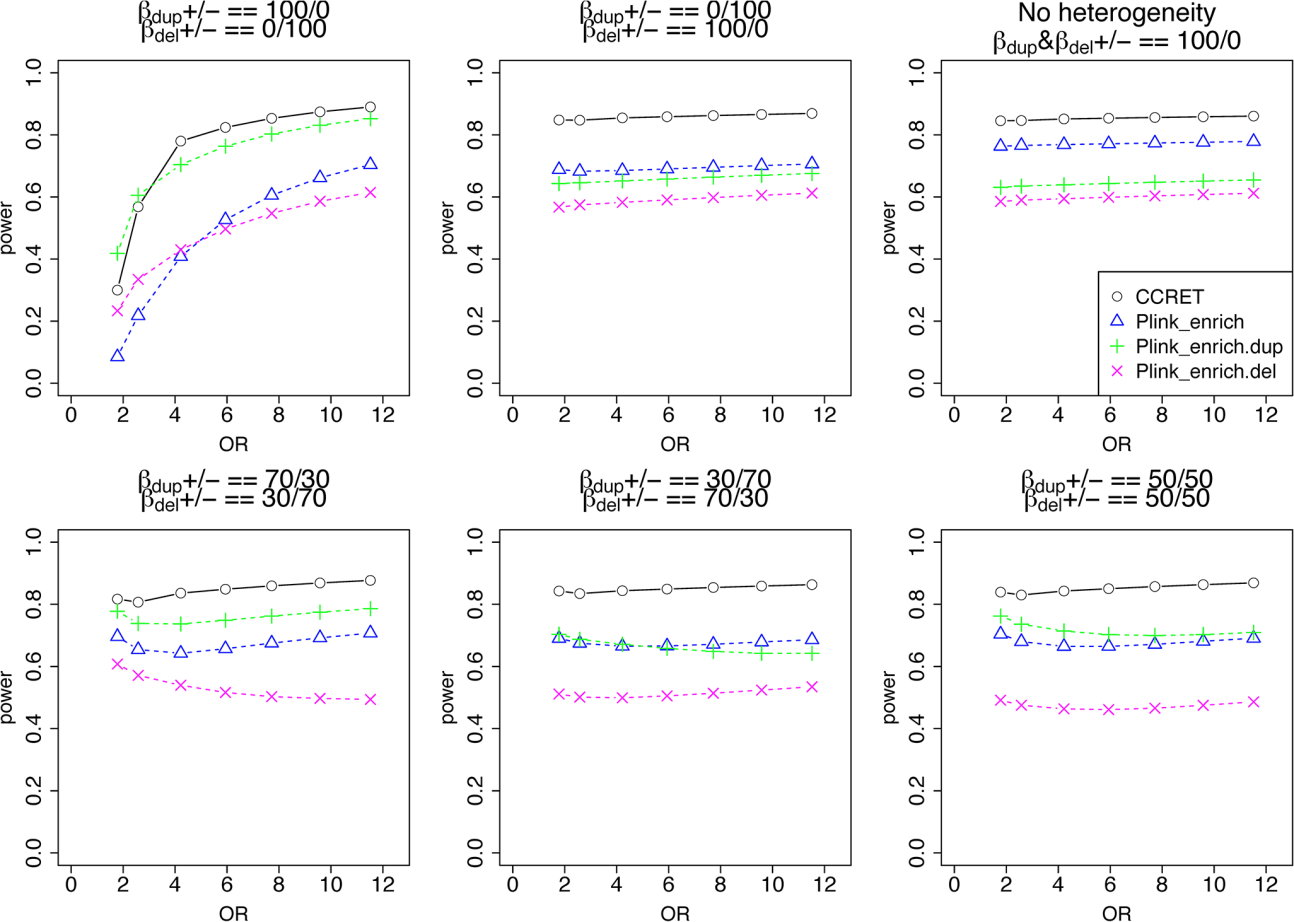
Power comparison between CCRET and PLINK 2-sided tests for simulation II-A: between-locus heterogeneity of the GI simulation, under 6 heterogeneity models as detailed in section “Simulation Design”. Black **O** line: CCRET; Blue **Δ**: PLINK 2-sided test analyzing deletions and duplications combined; Green **+**: PLINK 2-sided test analyzing only duplications; Magenta **x**: PLINK 2-sided test analyzing only deletions.

Table 2 shows that the type I error rates were around the nominal level for PLINK.enrich and CCRET, although CCRET was slightly conservative. Fig 6 suggests a consistent power gain of CCRET over PLINK.enrich, PLINK.enrich.dup and PLINK.enrich.del across all heterogeneity models. The consistent power gain in CCRET, which was not observed in the dosage simulation, was perhaps because the signal to noise ratio (SNR) in the GI simulation was smaller than that in the dosage simulation. Specifically, the SNR was 7.8% for this simulation but 34.1% in the dosage simulation. In SNP analysis, it has also been found that fixed effects approaches tend to be less powerful than random effects approaches when a high proportion of non-causal loci exist [19, 20]. Among the PLINK methods, PLINK-del has the lowest power in most of the scenarios, which is likely because in the causal genes, there are more duplication events intersecting the DupOnly causal genes than deletion events intersecting the DelOnly causal genes.

**Table 2:** Type I error rates for evaluating GI effects (nominal alpha = 0.05).

Model	CCRET	PLINKenrich	PLINK enrich.dup	PLINK enrich.del
(A) Between-locus heterogeneity	0.041	0.044	0.051	0.042
(B) Within-locus heterogeneity	0.043	0.051	0.052	0.043

#### Simulation II-B: within-locus heterogeneity of the GI simulation

**Fig 7:**
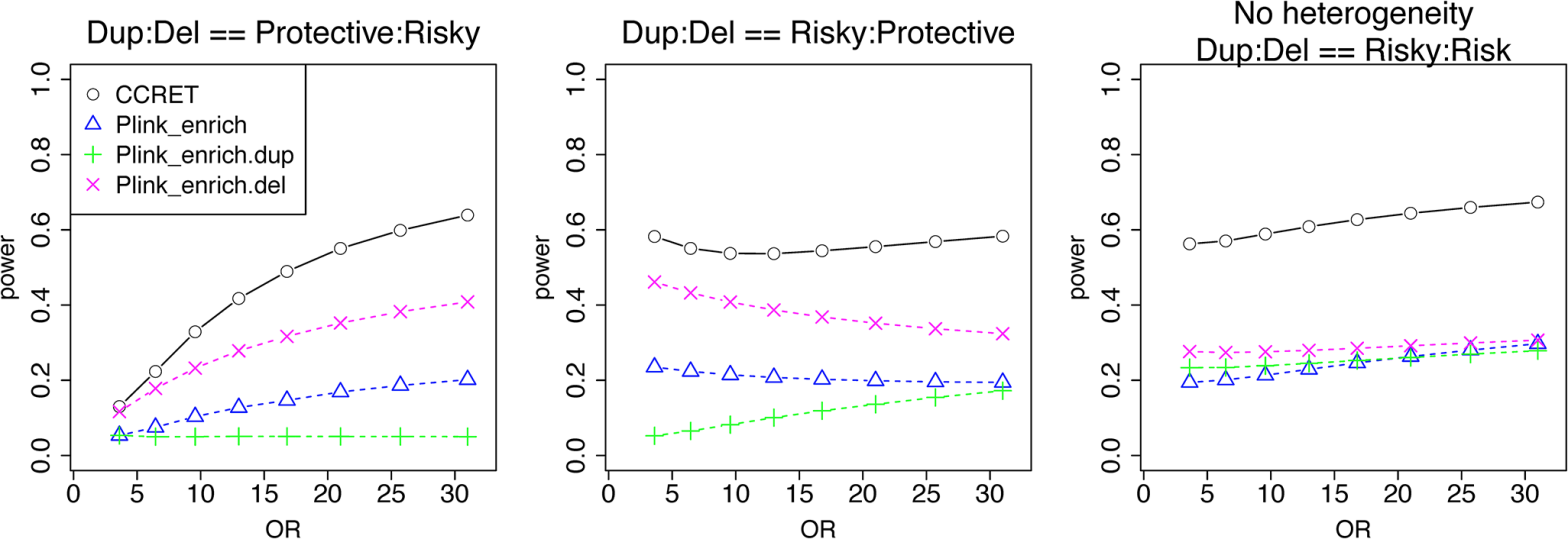
Power comparison between CCRET and PLINK 2-sided tests for simulation II-B: within-locus heterogeneity of the GI simulation, under 3 heterogeneity models as detailed in section “Simulation Design”. Black **O** line: CCRET; Blue **Δ**: PLINK 2-sided test analyzing deletions and duplications combined; Green **+**: PLINK 2-sided test analyzing only duplications; Magenta **x**: PLINK 2-sided test analyzing only deletions.

We used the 6 “DupDel” genes as causal and considered 3 heterogeneity models, i.e., (Dup, Del) = (P,R), (R,P) and (R,R) (no heterogeneity). We set a constant effect size for all casual loci and considered OR ranging between 3 and 50, chosen based on empirical evidences [4, 5]. The type I error rates (Table 2) were around the nominal level for PLINK.enrich methods and CCRET. The power results (Fig 7) suggest a consistent power gain of CCRET over PLINK.enrich based methods. The SNR was 1.2% for II-B and 2.2% in the I-B dosage simulation. In Fig 7, PLINK.del had the highest power among PLINK tests since, among the 8 DupDel causal genes, there were more deletions than duplications. Note that we used the same aggregate functions as PLINK.enrich to model the background CNVs (i.e., dosage and size) in CCRET’s GI model. Therefore the CCRET’s GI model was the same as the PLINK.enrich model except the GI effect was modeled using a random effect. Consequently, the power gain in CCRET can be attributed to the use of random effects modeling of the GI effect.

### Data application

We applied CCRET to CNV data from the Swedish schizophrenia study [36] and compared its performance to that of PLINK. The Swedish schizophrenia study used a case-control sampling design. Genotyping was done in six batches (Sw1-6) at the Broad Institute using Affymetrix 5.0 (3.9%, Sw1), Affymetrix 6.0 (38.6%, Sw2-4), and Illumina OmniExpress (57.4%, Sw5-6). We applied PennCNV [34] to generate CNV calls using the same protocol as we did for samples from the TwinGene project. After stringent quality control, we obtained a high-quality rare CNV (frequency <1%, size >100 Kb) dataset in 8,547 subjects (3,637 cases with schizophrenia and 4,820 controls) [36]. Further details on quality control can be found in ***S1 Text***. Previous analyses of this data indicated significant associations of large rare CNVs with schizophrenia risk for both genomewide dosage effects and GI effects of selected gene sets; and the associations were stronger when restricting to the largest categories (e.g. size > 500 kb) [9, 12]. CNVs with size >500 kb may be relatively more homogenous than CNVs with size > 100kb in the effects on disease risk as more causal genes may be impacted [9, 12].

We obtained the CNV data in PLINK format and converted it to the CCRET-format matrixes (“DS”, “Len”, “GI”). We verified that they stored identical information (i.e., the same total CNV numbers, average CNV lengths, and counts of intersected genes for each individual). We used the polynomial kernel with *d* = 1 and fit CCRET models (2) and (4) to evaluate the dosage effects and GI effects, respectively. The CCRET p-values were obtained using the Davies method [37]. We started by examining CNVs of size greater than 100kb, using CCRET and the 2-sided PLINK methods; and then repeated the analysis using CNVs of size greater than 500kb. In all analyses, we adjusted for the batch effect by including a batch indicator [9, 12]. As each genotyping batch used one specific type of arrays, controlling for genotyping batch effect simultaneously controlled for the difference in genotyping array. The comparison results are summarized in Table 3 for dosage effects and Table 4 for GI effects.

**Table 3:** Test p-values for evaluating dosage effects based on schizophrenia data.

Size	Type	*#CNVs total*	*Case CNV rate*	*ControlCNV rate*	*Case/controlratio*	*#CNVRstotal*	*#CNVRs with Dup*.*only*	*#CNVRs with Del*.*only*	*#CNVRs with DupDel*	*Pval_PLINK*	*Pval_CCRET*
>100Kb	DUP & DEL	8320	0.996	0.975	1.022	1853	1022 (55%)	517 (28%)	314 (17%)	0.597	0.002
	DUP	5070	0.599	0.600	0.997		1383			0.966	
	DEL	3250	0.397	0.375	1.061			897		0.192	
>500kb	DUP & DEL	840	0.110	0.091	1.199	300	202 (67%)	57 (19%)	41 (14%)	0.013	0.031
	DUP	617	0.080	0.068	1.175		246			0.062	
	DEL	223	0.030	0.024	1.267			99		0.085	

DUP: duplications. DEL: deletions. Case (or control) CNV rate = the total number of CNVs in cases (or controls) divided by the total number of cases (or controls). Case/control ratio = Case CNV rate divided by control CNV rate. CNVR: copy number variation region. Pval_PLINK: 2-sided p-values based on 10,000 permutations and permuting phenotype labels within genotyping batches (asymptotic p-values were similar). Pval_CCRET: 2-sided p-values based on the Davies (1980) method.

**Table 4:** Test p-values for evaluating GI effects based on schizophrenia data.

Gene-sets		CNVs	*CNVs > 100 Kb*						*CNVs > 500 Kb*					
Name (source)	#Genes	Type	*#Genes any CNV*	*#GenesDup*.*only*	*#GenesDel*.*only*	*#GenesDupDel*	*Pval PLINK*	*Pval CCRET*	*#Genesany CNV*	*#Genes Dup*.*only*	*#GenesDel*.*only*	*#GenesDupDel*	*Pval PLINK*	*Pval CCRET*
FRMP targets (Darnell et al.)	810	DUP & DEL	228	137	49	42	0.001	0.015	69	43	14	12	0.004	0.0006
		DUP		179			0.002			55			0.028	
		DEL			91		0.377				26		0.138	
PSD (Kirov et al)	668	DUP & DEL	153	100	27	26	0.110	0.018	55	29	14	12	0.002	0.008
		DUP		126			0.188			41			0.009	
		DEL			54		0.395				27		0.213	
PSD/mGluR5 (Kirov et al)	38	DUP & DEL	9	4	3	2	0.149	0.019	3	1		2	0.015	0.004
		DUP		6			0.225			3			0.048	
		DEL			5		0.622				2		0.295	
PSD/NMDAR (Kirov et al)	61	DUP & DEL	20	12	4	4	0.016	0.0007	9	4	3	2	0.002	0.002
		DUP		16			0.033			6			0.009	
		DEL			8		0.328				5		0.157	
PSD/PSD-95 (Kirov et al)	65	DUP & DEL	16	8	4	4	0.007	0.046	6	1	3	2	0.021	0.203
		DUP		12			0.344			3			0.727	
		DEL			8		0.008				5		0.001	
Mental Retardation	503	DUP & DEL	106	66	26	14	0.234	0.016	35	25	5	5	0.004	0.002
		DUP		80			0.099			3			0.004	
		DEL			40		0.661				1		0.750	
Synaptic genes (Ruano et al)	718	DUP & DEL	249	158	45	46	0.093	0.013	97	6	2	17	0.019	0.007
		DUP		203			0.457			77			0.337	
		DEL			92		0.060				38		0.002	
Synaptic Proteome (G2Cdb)	1023	DUP & DEL	177	112	38	27	0.0003	0.002	72	44	17	11	0.003	0.016
		DUP		139			0.002			55			0.025	
		DEL			66		0.086				29		0.117	
Cytoplasm (Kirov et al)	266	DUP & DEL	50	36	11	3	0.021	0.011	12	8	2	2	0.007	0.002
		DUP		39			0.008			1			0.006	
		DEL			14		0.821				4		0.574	

DUP: duplications. DEL: deletions. #Genes any CNV = the total number of genes intersected (≥1bp) by any CNV. #Genes Dup.only (or Del.only) = the total number of genes intersected (≥1bp) by duplications-only (or deletions-only). #Genes DupDel = the total number of genes intersected (≥ 1bp) by both duplications and deletions. Pval_PLINK: 2-sided asymptotic p-values based on Raychaudhuri et al (2010). Pval_CCRET: 2-sided p-values based on the Davies (1980) method.

For dosage effects with CNVs that are >100kb, the results of PLINK.all and CCRET are different: PLINK.all (p-value 0.597) suggested no signal while CCRET had a significant p-value of 0.002. When focusing on CNVs that are >500kb, we replicated the literature finding of a significant association [9, 12] using both PLINK and CCRET: the p-value of PLINK.all is significant (p-value 0.013) while the p-value of CCRET is comparable (p-value 0.031). It seems that CNVs with size >500kb have relatively homogeneous dosage effects on the schizophrenia risk, where the case/control ratios of CNV rate are in the same directions for deletions-only and duplications-only (both >1.1, indicating an excess burden in cases). On the other hand, CNVs with size >100kb could have heterogeneous dosage effects, where the case/control ratios of CNV rate are in different directions for deletions-only (>1) and duplications-only (≤1). Consequently, PLINK.all had a significant result with >500kb CNVs but insignificant results with >100kb. In contrast, CCRET, due to its robustness with respect to the heterogeneity, yielded significant p-values for both types of CNVs. The results are not unexpected based on our findings in the simulations, where we observed that when CNVs have relatively homogeneous effects (which may occur in the case of CNVs >500kb in the real data), the best PLINK method can be more powerful than CCRET; when CNVs have heterogeneous effects (which may occur in the case of CNVs >100kb in the real data), CCRET is more powerful than PLINK.

For GI effects, we analyzed nine gene sets where significant enrichments of large rare CNVs in schizophrenia cases have been previously reported [12]. Further details on these gene sets can be found in ***S1 Text***. For most gene sets except for PSD-95, a similar pattern was observed with the GI analysis as with the dosage analysis: With CNVs > 500kb, we replicated the literature finding of significant associations [12] using both PLINK.enrich and CCRET. With CNVs > 100kb, we obtained different results between PLINK.enrich and CCRET,where CCRET often yielded significant p-values while PLINK.enrich did not. These results are not unexpected based on our findings in the simulations. For PSD-95, we observed significant results using PLINK.enrich for both CNVs > 100kb and CNVs > 500kb but insignificant results using CCRET. These results perhaps were influenced by the potentially high signal-to-noise-ratio in this small gene set (i.e. high proportion of causal loci) and relatively more homogenous CNV effects, which favors fixed effects collapsing methods over random effects methods.

## Discussion

CNVs play an important role in disease etiology [4, 5]. While it is more informative to examine CNVs in the aggregate due to sparse events or modest marginal effects, current polygenic methods are mainly designed for bi-allelic SNPs and do not fully address the CNV-specific challenges. The challenges include the multiple features of a CNV affecting phenotypes, the non-biallelic nature of CNV polymorphisms, and etiological heterogeneity within and between loci. In this work, we introduce CCRET, a random effects method for CNV collapsing analysis that accommodates the poly-allelic nature of CNVs, models multiple CNV features and accounts for etiological heterogeneity. Simulation and real data analyses suggest that CCRET has stable performance across different scenarios of heterogeneity, and has a comparable or better power when compared to PLINK collapsing methods. The largest power gain tends to occur when heterogeneity pattern are complex, e.g., a mixture of risk-associated and protective effects observed within a locus or within a certain CNV type (duplication or deletion). The average running time for performing CCRET analysis with 4000 individuals is 27.5 seconds on an Intel Xeon 3.06 GHz machine with 64 Gb RAM.

With CCRET, we address the etiological heterogeneity by retaining the “locus”-specific effects and “allele”-specific effects during collapsing. To retain the locus-specific effect during collapsing, we define a locus unit for each CNV feature (e.g., CNVRs for dosage and genes for gene intersection); based on the locus definition, we quantify similarity shared between an individual pair for each locus and then aggregate the similarity information across loci. Because the multi-locus information is aggregated through a sum of genotype similarity instead of a sum of genotypes, loci with opposite effects do not cancel each other out. To retain allele-specific effect during collapsing, we factorize the allele values recorded in the design matrixes before calculating similarity. Factorizing the allele values avoids the need of dichotomizing the polymorphisms of a CNV feature and gains robustness against within-locus heterogeneity (as alleles with opposite effects do not need to be lumped together).

For GI analysis, we use genes instead of CNVRs as the locus unit. If CNVRs are used, the entry of the design matrix would record the number of genes intercepted by a certain CNV in the CNVR. Such data scoring may not be ideal when different intersected genes have different etiological effects. Hence we use genes as the locus unit so that the entry of the design matrix records the type of CNVs intersecting a gene. This allows us to model the gene-specific effects as well as the effects of the specific CNV type that intersects the gene.

Currently, there is no clear consensus on how to define CNVRs. One typical definition of CNVR is based on forming clusters of individual segments with some arbitrary amount of overlapping (e.g., 1 base pair overlap, 50% reciprocal overlap), and then allele frequency is computed for each CNVR. The choice of overlapping threshold could impact the formation of heterogeneous clusters that contain segments of variable sizes and breakpoints, each of which only overlap by a few base pairs but which may be disease relevant. For example, rare *NRXN1* deletions are associated with schizophrenia and show variable breakpoints and lengths among schizophrenia cases [38]. Such heterogeneous clusters of rare CNVs can occur frequently with high-resolution CNV detection technologies (e.g., high-density microarray and high-throughput sequencing); therefore defining CNVRs requires care [1, 4, 5]. In this work, we used the most inclusive threshold of 1 base-pair overlap to define CNVRs. Future study could account for CNVRs overlapping pattern by including them as fixed-effect covariates.

The simulation analyses show that, while sensitive to the underlying effect mechanism, the best PLINK (fixed effects) tests can be more powerful than CCRET when the effects of CNVs are homogeneous (such as the effects of >500kb CNVs in the real data analysis). The results agree with the findings in the SNP collapsing analysis; that is, the fixed effects approaches, which use the total event count to summarize the variant information within the target region, are the optimal methods when the majority of the variants in the region affect the phenotype in the same direction and with similar magnitude. On the other hand, when there exist non-causal variants (e.g., the effects of >100kb CNVs in the real data analysis) or a mixture of risk-associated and protective variants, random effects approaches are optimal because of their ability to account for effect heterogeneity. Taking together, we recommend that researchers apply both PLINK (fixed effects) and CCRET (random effects) in real-world rare CNV analysis because the underlying mechanisms of genetic effects are typically unknown.

Further, one future direction in which CCRET could be improved is to consider a hybrid approach, such as the SKAT-O test for rare SNP analysis [39]. Such a hybrid approach can retain the robustness of the random effects approach while retaining the test power of the fixed effects approach when the CNVs have homogeneous effects.

In the current work, we modeled the CNV feature of interest using random effects and the background CNV features using fixed effects. Alternatively, one can use random effects to model all CNV features; under this “fully random effects” model, the overall effect of each CNV feature is captured by a variance component. However, the calculation of the test statistics can be extremely computationally intensive, especially with large samples and non-normal traits, because it involves estimating several nuisance variance components. Similar computational concerns have also been encountered in GLMM based approaches for gene-environment interactions [40, 41]. We are exploring possible low-rank approximations to the CNV kernel matrixes using kernel principal component analysis [42] to reduce the computational burden in a fully random effects model.

To date, significant associations of rare CNVs with psychiatric disorders have been limited to the largest CNVs (e.g. >500 kb) [1, 4, 5]. In this work, the significant results with CNVs > 100kb obtained by CCRET are intriguing. There have been increasing evidences that smaller CNVs may contribute to the risk of psychiatric disorders [43, 44], although such associations have been challenging to detect because analytic methods are under developed. Improvement in CNV detection technologies will increase our ability to detect smaller CNVs (e.g., <20 kb), for which etiological heterogeneity can be more frequently encountered. The CCRET method could be important in analyzing smaller CNVs given its robustness in a variety of heterogeneous scenarios.

As we move ahead, success will increasingly depend on our ability to integrate all classes of genetic variation into a more complete disease model, including joint analysis of SNPs and CNVs [45]. For example, in schizophrenia genetics, multiple lines of genomic inquiry–genome-wide screens for rare CNVs, common SNPs, and rare exonic variation–are converging on similar sets of pathways and/or genes [12, 35, 36, 46]. The CCRET method may open a door for joint analysis as various variant types can be incorporated and simultaneously modeled under a random effects framework.

## Materials and Methods

### Ethics statement

All procedures were approved by ethical committees at the Karolinska Institutet in Sweden and at the University of North Carolina at Chapel Hill in the US, and all subjects provided written informed consent (or legal guardian consent and subject assent).

### Input data format

As PLINK (version 1.07) is the standard software for CNV analysis from case/control data, we assume a PLINK-format CNV file as the source data, which lists the base pair position (start and end) and copy number (or dosage coded as 0,1,3,4+) of individual CNV segments. We create three input matrixes to store the different features of CNVs, i.e., dosage (“DS”), length (“Len”), and gene intersection (“GI”). For dosage and length matrixes, we first form the CNV region (CNVR) as the “locus” unit by clustering CNV segments using ≥1bp overlap from the PLINK format file. The (*i*, *m*)-entry of the DS matrix indicates the copy number (0, 1, 2, 3, 4+) of the CNV segment for subject *i* at CNVR *m*, and the (*i*, *m*)-entry of the Len matrix indicates the corresponding segment size in the DS matrix. The use of CNVRs allows us to keep track of CNVR-specific effects and to account for heterogeneity between and within CNVRs in the collapsing analysis.

For the gene intersection (“GI”) matrix, instead of using CNVRs as we did for dosage and length, we use genes as the collapsing units to keep track of the effect of different genes when they are interrupted by CNVs. We obtained the coordinates of the genes of interest (http://genome.ucsc.edu/), and then create the GI matrix where the (*i*, *m*)-entry indicates whether gene *m* of subject *i* is intersected (≥1bp overlapping) by a CNV and the corresponding CNV type (i.e., 0 for no intersection, 1 for intersection by a deletion, and 2 for intersection by a duplication). The genes of interest can be a collection of all protein coding genes or a collection of genes in a specific pathway. Using the gene as the locus unit allows us to evaluate the heterogeneous effects between and within genes in specific pathways. Alternatively, an exon could be used as the observation unit.

CCRET can handle CNVs called from both microarray and sequencing data. CNV files generated by CNV-calling algorithms from sequencing data are either in the Variant Call Format (VCF), such as those used by the 1000 Genomes project [3, 47], or in VCF-like but algorithm-specific output format (e.g., DATA.xcnv produced by the XHMM [48, 49] software from exome sequencing data). With CCRET, its data preparation pipeline provides scripts to convert any input CNV files first to PLINK-format CNV files as the source data and then to CCRET-specific CNV input matrixes (DS, Len, GI) for use in random effects modeling.

### CCRET method

For subject *i*, let *Y*_*i*_ be a continuous or binary trait, *X*_*i*_ be a *p* × 1 covariate vector including the intercept, and $Z_{i}^{f}=\left[Z_{i1}^{f},\cdots ,Z_{iM_{f}}^{f}\right]$ be a *M*_*f*_ × 1 design vector of feature *f* in a certain genomic region, e.g., whole genome, pathway or certain type of genes. For *f* = *DS* or *Len*, the design vector length, *M*_*f*_, is the number of CNVRs in the targeted genomic region; for *f* = *GI*,*M*_*GI*_ is the number of genes in the targeted genomic region.

Suppose *Y*_*i*_ follows a distribution from the exponential family with density *f*_*Y*_(*Y*_*i*_;*θ*_*i*_,*ϕ*) = exp[{*θ*_*i*_ *Y*_*i*_ − *b*(*θ*}/{*ϕv*_*i*_}] + *c*(*y*_*i*_,*ϕ*), where *θ*_*i*_ is the canonical parameter with *θ*_*i*_ = *g*(*μ*_*i*_) with *g*(⋅) being a known link function, $\mu_{i}=E(Y_{i}\;|\;{X_{i},Z}_{i}^{DS},Z_{i}^{GI},Z_{i}^{Len})$, *b*(⋅) and *c*(⋅) are known functions, *ϕ* is a dispersion parameter, and *v*_*i*_ is a known weight. The mean and variance of *Y*_*i*_ satisfy *μ*_*i*_ = *b*′(*θ*_*i*_) and $V(Y_{i}\;|\;{X_{i},Z}_{i}^{DS},Z_{i}^{GI},Z_{i}^{Len})=\phi v_{i}^{-1}b\prime \prime (\theta_{i})$ where $b\prime (\theta_{i})=\frac{\partial }{\partial \theta_{i}}b(\theta_{i})$ and $b\prime \prime (\theta_{i})=\frac{\partial }{\partial \theta_{i}}b\prime (\theta_{i})$. We posit the following model to study the effects of CNV features on the trait values:

$$g(\mu_{i})=\gamma_{0}X_{i}+h_{DS}\left(Z_{i}^{DS}\right)+h_{GI}\left(Z_{i}^{GI}\right)+h_{Len}\left(Z_{i}^{Len}\right),$$ (1)

where the covariate effects are modeled with effect size vector ${\gamma_{0}}_{p\times 1}$, and *h*_*f*_(⋅) with *f* ∈ {*DS*, *GI*, *Len*} is a smooth function that models the effect of CNV feature *f*. There are many possible choices for the functions *h*_*f*_(⋅). For example, one may set $h_{f}(Z_{i}^{f})=\sum_{m=1}^{M_{f}}{\beta_{f,m}Z}_{im}^{f}$, allowing each CNVR to have its own effect *β*_*f*,*m*_, therefore maximizing its ability to capture heterogeneity. This model corresponds to a classic linear regression but suffers from low power due to large degrees of freedom and sparse information. In order to reduce the degrees of freedom, one may impose a random effects model by assuming that the individual effects follow a normal distribution *β*_*f*,*m*_ ∼ *N*(0,*τ*_*f*_). Alternatively, Raychaudhuri et al.[27] (PLINK methods) considered the aggregated functions $h_{DS}(Z_{i}^{DS})=\beta_{DS}\times \sum_{m=1}^{M_{DS}}I\{Z_{im}^{DS}\neq 2\}$ (where $\sum_{m=1}^{M_{DS}}I\{Z_{im}^{DS}\neq 2\}$ is the total number of CNVs of subject *i*) and ${h_{GI}(Z_{i}^{GI})=\beta }_{GI}\times \sum_{m=1}^{M_{GI}}I\{Z_{im}^{GI}\neq 0\}$ (where $\sum_{m=1}^{M_{GI}}I\{Z_{im}^{GI}\neq 0\}$ is the number of genes intersected by CNVs for subject *i*), which nicely amplify the information content and avoid the problem of dimensionality by testing the averaged/common effect.

With CCRET, to avoid the dimensionality problem, we propose to model the covariates and background CNV features (measured from whole genome) using fixed effects, such as by setting *h*_*f*_(⋅) as those considered in Raychaudhuri et al [27], but to model the CNV feature of interest (measured from the genomic regions of interest) using random effects. Specifically, when evaluating the effect of CNV feature *f*, we set $h_{f}(Z_{i}^{f})\equiv h_{i}^{f}$, where $h_{i}^{f}$ represents the subject-specific effect of multi-locus CNVs with feature *f* and is assumed to be random. Modeling the targeted CNV feature using random effects can capture the locus-specific details and account for between-locus and within-locus etiological heterogeneity when collapsing the information across different CNV regions. Treating the background CNV features as fixed effects can greatly boost the computational efficiency by bypassing the need to estimate the nuisance variance components, which can be burdensome with non-normal traits. As in the random effects approaches for SNP analyses (e.g., C-alpha [15], SKAT [16], SimReg [17, 18]), we assume that $h^{f}={\left(h_{1}^{f},\cdots ,h_{n}^{f}\;\right)}^{T}∼N(0,\tau_{f}\;K_{f})$ where $K_{f}={\left\{{\;K}_{f}(Z_{i}^{f},Z_{j}^{f})\;\right\}}_{i,j=1}^{n}$ and *K*_*f*_(∙,∙) is a distance metric that quantifies the similarity between subject *i* and subject *j* based on CNV feature *f* in the targeted genomic region. In other words, the CNV information of feature *f* in the targeted region is first summarized by genetic similarity, which is then used to depict the covariance between CNV effects $h_{i}^{f}$ and $h_{j}^{f}$. The aggregate CNV effect of feature *f* can be evaluated by examining the significance of the variance component *τ*_*f*_ (i.e., testing *H*_0_: *τ*_*f*_ = 0).

### CCRET’s connection with other random-effects collapsing methods

The proposed CCRET model has a direct connection with kernel machine regression [16, 50, 51] and gene-trait similarity regression [17, 18] because both kernel machine and similarity regressions have a mixed model representation. Specifically, under the kernel machine framework, our CCRET model is equivalent to specifying the CNV feature of interest, *h*_*f*_(⋅), through a linear combination of kernel functions *K*_*f*_(∙,∙)’s. That is, $h_{f}(Z_{i}^{f})=\sum_{j=1}^{n}\alpha_{j}^{f}\times K_{f}(Z_{i}^{f},Z_{j}^{f})$ with $\alpha_{j}^{f}$ being the unknown parameter (the dual representation), or the equivalent basis representation, $h_{f}(Z_{i}^{f})=\sum_{l=1}^{L}{\eta_{l}^{f}\times \phi }_{l}^{f}(Z_{i}^{f})$, where ${\{\phi }_{1}^{f}(Z_{i}^{f}),\cdots ,\phi_{L}^{f}(Z_{i}^{f})\}$ is a set of the orthogonal basis functions spanning the functional space specified by *K*_*f*_(∙,∙) and $\eta_{l}^{f}$ is the unknown parameter. Under the similarity regression framework, where the genetic effect is assessed by the model significance that regresses trait similarity on genetic similarity, the variance component, *τ*_*f*_, in our CCRET model is equivalent to the regression coefficient of genetic similarity quantified by the distance metric *K*_*f*_(∙,∙).

### Quantification of CNV similarity between two individuals

From the connection with other random-effects collapsing methods, we see that one can determine how to model the multi-locus CNV information by selecting the desired metrics (kernels) to quantify similarity between subjects *i* and *j*. To fix the idea, consider the commonly used *d*-th order polynomial function, i.e., $K_{f}(Z_{i}^{f},Z_{j}^{f})={\left(1+\sum_{m=1}^{M_{f}}{{w_{m}\times Z}_{im}^{f}\times Z}_{jm}^{f}\right)}^{d}$, where *w*_*m*_ is the pre-specified weight for locus *m* based on, for example, some inverse function of the allele frequencies if CNVs of different frequency ranges are evaluated together. When *d* = 1, this corresponds to the model with only main effects, i.e., $h(Z_{i}^{f})=\sum_{m=1}^{M_{f}}{\eta_{m}^{f}\times Z}_{im}^{f}$; when *d* = 2, this corresponds to a model with linear and quadratic main effects as well as two-way interactions, i.e., $h(Z_{i}^{f})=\sum_{m=1}^{M_{f}}{\eta_{1m}^{f}\times Z}_{im}^{f}+\sum_{m=1}^{M_{f}}{\eta_{2m}^{f}\times (Z}_{im}^{f}{)}^{2}+\sum_{l<m}^{M_{f}}{\gamma_{lm}^{f}\times Z}_{il}^{f}{\times Z}_{im}^{f}$. For copy number dosage, it may not be sensible to use directly $Z_{im}^{DS}$ and plug it in to the kernel function because both $Z_{im}^{DS}<2$ and $Z_{im}^{DS}>2$ deviate from normal copy number, while directly plugging $Z_{im}^{DS}$ implies a dosage effect with copy number 0 as baseline. To resolve this issue, when quantifying similarity based on CNV dosage, we suggest to first covert $Z_{im}^{DS}$ to a factorized design vector $G_{im}^{DS}$. For example, for a dosage ranging from 0 to 4, we define a length-3 design vector $G_{im}^{DS}=[1\;0\;0]$ for dosage < 2, [0 1 0] for dosage = 2 and [0 0 1] for dosage > 2; then $K_{f}(Z_{i}^{DS},Z_{j}^{DS})={\left(1+\sum_{m=1}^{M_{f}}{{w_{m}\times G}_{im}^{DS}}^{T}G_{jm}^{DS}\right)}^{d}$. The factorized design vector naturally accommodates the multi-categorical nature of dosage or GI. For discrete SNP data, one commonly used distance metric is the identity-by-state (IBS) metric, which is the proportion of alleles shared between two subjects in the targeted region. For CNV dosage, the IBS score at locus *m* becomes $G_{im}^{T}G_{jm}$ and is indeed the linear kernel.

### Evaluating dosage effects

Below we illustrate the details of the proposed CCRET method using CNV dosage effects as an example, where we aim to assess the dosage effect while adjusting for the effects of CNV length and CNV gene counts. Following Raychaudhuri et al. [27], we define ${\tilde{Z}}_{i}^{GI}$ as the total number of genes that are intersected (i.e., including disrupted and overlapped) by the CNVs for subject *i*; define ${\tilde{Z}}_{i}^{Len}$ as the mean CNV length in kb of subject *i*. For those subjects with no CNVs (i.e., ${\tilde{Z}}_{i}^{Len}=0$), their ${\tilde{Z}}_{i}^{Len}$ values are set to be the mean of the non-zero ${\tilde{Z}}_{i}^{Len}$’s. For dosage analysis, we rewrite Model (1) as

$$g(\mu_{1i})=\gamma_{1}X_{i}+\beta_{Len}{\tilde{Z}}_{i}^{Len}+\beta_{GI}{\tilde{Z}}_{i}^{GI}+h_{i}^{DS},$$ (2)

where $h^{DS}={\left(h_{1}^{DS},\cdots ,h_{n}^{DS}\right)}^{T}∼N(0,\tau_{DS}\;K_{DS})$ and *K*_*DS*_ is an *n* × *n* matrix with $K_{DS}(i,j)=K_{DS}(Z_{i}^{DS},Z_{j}^{DS})$. Under Model (2), the dosage effect can be evaluated by testing $H_{0}^{DS}:\tau_{DS}=0$. The incorporation of the background CNV features when assessing dosage effect is mainly for reducing the unexplained variance in the model and hence enhancing the detecting power. Using a similar derivation as Tzeng et al. [17], we construct a score-based test statistic to assess the dosage effect by considering a matrix presentation of Model (2):

$$g\left(\mu_{1}\right)=X\gamma_{1}+{\tilde{Z}}^{Len}\beta_{Len}+{\tilde{Z}}_{}^{GI}\beta_{GI}+h^{DS},$$ (3)

where *μ*_1_ = (*μ*_11_,⋯,*μ*_1*n*_)^*T*^, *X* = (*X*_1_,⋯,*X*_*n*_)^*T*^, ${\tilde{Z}}_{}^{Len}={\left({\tilde{Z}}_{1}^{len},\cdots ,{\tilde{Z}}_{n}^{len}\right)}^{T}$, and ${\tilde{Z}}_{}^{GI}={\left({\tilde{Z}}_{1}^{GI},\cdots ,{\tilde{Z}}_{n}^{GI}\right)}^{T}$. Using a very similar derivation of Tzeng and Zhang [52] and Tzeng et al. [17], it can be shown that the score test statistic is given as

$$T_{DS}=\frac{{\left(Y-\mu_{1}\right)}^{T}\Delta_{1}W_{1}K_{DS}W_{1}\Delta_{1}{\left(Y-\mu_{1})\right)}^{}}{2}\;{|}_{\tau_{DS}=0,\;\mu_{1}=\hat{\mu_{1}},\;\phi_{1}=\hat{\phi_{1}}}$$

where $\mu_{1}=g^{-1}(\tilde{X_{1}}\theta_{1})$ with $\tilde{X_{1}}=(X,{\tilde{Z}}^{Len},{\tilde{Z}}^{GI})$ and *θ*_1_ = (*γ*_1_, *β*_*Len*_, *β*_*GI*_)^*T*^, Δ_1_ = *diag*{*g*′(*μ*_1*i*_)}, and *W*_1_ = *diag*{*w*_1*i*_}, with $w_{1i}={\left\{\phi_{1}m_{1i}^{-1}b\prime \prime (\theta_{1i})){\left[g\prime (\mu_{1i})\right]}^{2}\right\}}^{-1}$. Estimate $\hat{\theta_{1}}$ is the maximum likelihood estimate (MLE) of *θ*_1_ under *H*_0_, and $\hat{\phi_{1}}$ is the restricted maximum likelihood (REML) type of estimate of *ϕ*_1_ under *H*_0_. As shown in Tzeng and Zhang [52], and Tzeng et al. [17], *T*_*DS*_ asymptotically follows a weighted chi-squared distribution, i.e., $T_{DS}\approx \sum_{l=1}^{C}\lambda_{1l}\chi_{1,l}^{2}$, where $\lambda_{1l}$’s are the non-zero eigenvalues of $W_{1}^{-\frac{1}{2}}P_{1}K_{DS}P_{1}W_{1}^{-\frac{1}{2}}$ and $P_{1}=W_{1}-W_{1}\tilde{X_{1}}{\left({\tilde{X_{1}}}^{T}W_{1}\tilde{X_{1}}\right)}^{-1}{\tilde{X_{1}}}^{T}W_{1}$. The corresponding *p*-values can be obtained by Davies’s methods (1980) [37] or by moment matching approaches as discussed in [53].

### Evaluating gene-intersection (GI) effects

A very similar procedure can be used to assess the gene-intersection effect while adjusting for the effects of CNV length and the total number of CNV events. Specifically, we consider

$$g\left(\mu_{2i}\right)=\gamma_{2}X_{i}+\delta_{Len}{\tilde{Z}}_{i}^{Len}+\delta_{DS}{\tilde{Z}}_{i}^{DS}+h_{i}^{GI},$$ (4)

where ${\tilde{Z}}_{i}^{DS}$ is the total number of CNV events of subject *i* in the whole genome, ${\tilde{Z}}_{i}^{Len}$ is as defined before, $h^{GI}={\left(h_{1}^{GI},\cdots ,h_{n}^{GI}\right)}^{T}∼N(0,\tau_{GI}\;K_{GI})$ and *K*_*GI*_ is an *n* × *n* matrix with $K_{GI}(i,j)=K_{GI}(Z_{i}^{GI},Z_{j}^{GI})$. By a similar derivation as in the dosage analysis, the score statistics for testing = 0 can be obtained as:

$$T_{GI}=\frac{{\left(Y-\mu_{2}\right)}^{T}\Delta_{2}W_{2}K_{GI}W_{2}\Delta_{2}{\left(Y-\mu_{2}\right)}^{}}{2}\;{|}_{\tau_{GI}=0,\;\mu_{2}=\hat{\mu_{2}},\;\phi_{2}=\hat{\phi_{2}}}$$

where $\mu_{2}=g^{-1}(\tilde{X_{2}}\theta_{2})$ with $\tilde{X_{2}}=\left(X,{\tilde{Z}}^{Len},{\tilde{Z}}^{DS}\right)$ and *θ*_2_ = (*γ*_2_, *δ*_*Len*_, *δ*_*DS*_)^*T*^; Δ_2_, *W*_2_, $\hat{\theta_{2}}$, and $\hat{\phi }$ are defined in a fashion similar to the dosage test. The p-value of *T*_*GI*_ can also be obtained by Davies’s methods (1980) [37] or by moment matching approaches as discussed in [53], because $T_{GI}\approx \sum_{l=1}^{C}\lambda_{2l}\chi_{1,l}^{2}$, where the $\lambda_{2l}$’s are the non-zero eigenvalues of ${W_{2}}^{-\frac{1}{2}}P_{2}K_{GI}P_{2}{W_{2}}^{-\frac{1}{2}}$ and $P_{2}=W_{2}-W_{2}\tilde{X_{2}}{\left({\tilde{X_{2}}}^{T}W_{2}\tilde{X_{2}}\right)}^{-1}{\tilde{X_{2}}}^{T}W_{2}$.

### PLINK methods

We evaluated the performance of CCRET using the PLINK methods (version 1.07, http://pngu.mgh.harvard.edu/~purcell/plink/) as a benchmark. For dosage effect of the whole genome, we compared CCRET with the burden-style methods [9] as implemented in PLINK “—cnv-indiv-perm”. This method fits a regression model: (*μ*_*i*_) = *α*_0_ + *α*_*c*_ ∙ *c*_*i*_, where *c*_*i*_ is the total number of events that are of interest (e.g., deletion, duplication, duplication+deletion etc.) for individual *i*. The “—cnv-indiv-perm” evaluates the significance of *α*_*c*_ via a permutation procedure. The default option returns 1-sided empirical *p*-values, assuming that the events of interest are more common in cases than in controls (i.e. events increase risk). The default 1-sided tests have been commonly adopted in practice [8–12]. Adding the flag “—cnv-test-2sided” will return 2-sided empirical *p*-values, allowing that the events of interest might be more common either in cases or in controls. For the GI effect of a gene set, we compared CCRET with the enrichment-style method of Raychaudhuri et al [27], which is implemented as “—cnv-enrichment-test” test in PLINK. This method fits a logistic regression model: $g(\mu_{i})=\beta_{0}+\beta_{DS}∙{\tilde{Z}}_{i}^{DS}+\beta_{Len}∙{\tilde{Z}}_{i}^{Len}+\beta_{GI}∙g_{i}$, where ${\tilde{Z}}_{i}^{DS}$ and ${\tilde{Z}}_{i}^{Len}$ are as defined earlier, *g*_*i*_ is the total number of intersected genes in a predefined gene set and *β*_*f*_’s are regression coefficients. The “—cnv-enrichment-test” tests if *β*_*GI*_, the coefficient associated with GI counts, is significantly different from 0. The default option returns 2-sided asymptotic *p*-values, allowing that gene intersection might be more common either in cases or in controls.

### Implementation

Source code of CCRET is available at http://www4.stat.ncsu.edu/~tzeng/software.php.

## Supporting Information

S1 Text This section includes the detailed information of the datasets used in this study. (DOCX)
