# Supplementary material for: A New Method for Detecting Associations with Rare Copy-Number Variants
Source: PLoS Genet. 2015 Oct 2;11(10):e1005403. doi: 10.1371/journal.pgen.1005403 (PMC4592002; doi:10.1371/journal.pgen.1005403)
Supplement: S1 Text — (DOCX) [file pgen.1005403.s001.docx]

***Supporting Information***

A New Method for Detecting Associations with Rare Copy-Number Variants

Jung-Ying Tzeng,^1,2^ Patrik KE Magnusson,^3^ Patrick F Sullivan,^3,4^ The Swedish Schizophrenia Consortium^¶^, Jin P Szatkiewicz ^3^*

* Correspond with [jin_szatkiewicz@med.unc.edu](mailto:jin_szatkiewicz@med.unc.edu)

**Detailed Information of the Datasets Used in This Study**

Two datasets were used in this work: the data from the TwinGene project [1, 2] were used as the basis for simulation because of its cross-sectional sampling design; the data from the Swedish schizophrenia study [3], obtained from a case-control sampling design, were used to test the utility of the proposed method. Each datasets were described in further details below.

Ethics statement. All procedures were approved by ethical committees at the Karolinska Institutet in Sweden and at the University of North Carolina at Chapel Hill in the US, and all subjects provided written informed consent (or legal guardian consent and subject assent).

*The TwinGene project.* The Swedish Twin Registry [4-6] (STR) was first established in the late 1950s to study the importance of smoking and alcohol consumption on cancer and cardiovascular diseases and, at the present, the STR included 85,000 twin pairs born in Sweden. From this STR, the TwinGene project (conducted in 2004-2008) selected subjects born between 1911 and 1958 for genomic characterization. DNA was extracted from peripheral venous blood for all subjects. A total of 6,611 unrelated samples were genotyped on the Illumina OmniExpress beadchip at Uppsala using standard protocol. These samples included one member per monozygotic twin and a randomly selected member per dizygotic twin. Standard data normalization procedures and canonical genotype clustering files provided by Illumina were used to process the genotyping signals. The signal intensities, i.e., the Log R ratio (LRR) and B allele frequency (BAF), were exported from Illumina BeadStudio. A total of 72,881 SNP markers were mapped to the hg19 human genome assembly. CNV calling was performed using the PennCNV [7] software (version June 2011) using recommended model parameters and the genomic wave adjustment routine. PennCNV applies a hidden-Markov model to LRR and BAF and integrates information in population frequency of B allele (PFB).

*The Swedish Schizophrenia Study.* Details about subject ascertainment, diagnosis, validation, and genotyping quality control are described in Ripke et al [3]. All procedures were approved by ethical committees in Sweden and the US, and all subjects provided written informed consent. Cases with schizophrenia were identified using the Swedish Hospital Discharge Register which captures all public and private inpatient hospitalizations. Case inclusion criteria: ≥2 hospitalizations with a discharge diagnosis of schizophrenia, both parents born in Scandinavia, and age ≥18 years. Case exclusion criteria: hospital register diagnosis of any medical or psychiatric disorder mitigating a confident diagnosis of schizophrenia. Controls were selected at random from Swedish population registers, with the goal of obtaining an appropriate control group and avoiding “super-normal” controls that can cause substantial bias in psychiatric research. Control inclusion criteria: never hospitalized for schizophrenia or bipolar disorder, both parents born in Scandinavia, and age ≥18 years. DNA was extracted from peripheral venous blood for all subjects. Genotyping was done in six batches (Sw1-6) at the Broad Institute using Affymetrix 5.0 (3.9%, Sw1), Affymetrix 6.0 (38.6%, Sw2-4), and Illumina OmniExpress (57.4%, Sw5-6). All genomic locations are given in NCBI build 37/UCSC hg19 coordinates. We applied PennCNV to generate CNV calls using the same protocol as we did for samples from the TwinGene project.

*Preparation of high-quality rare CNV datasets.* The CNV calling algorithm (PennCNV) was optimized to identify rare CNVs (<1%) but spurious CNVs can result from algorithm artifacts. Next, we extracted CNVs from the 22 autosomes and applied identical quality control (QC) procedures to each of the aforementioned datasets. The QC included removal of low-confidence CNVs with confidence scores < 10, spanning < 10 probes, or < 100 kb in length followed by removal of CNVs with > 50% reciprocal overlap with large genomic gaps (e.g., centromeres) or regions subject to rearrangement in white blood cells. We annealed adjoining CNVs that appeared to be artificially split by the CNV calling software by recursively joining CNVs if the called region is ≥ 80% of the entire region to be joined. We excluded subjects whose genotyping arrays had excessive noise (probe intensity variance or genomic “waviness” exceeding platform specific thresholds) or excessive CNV calls scattered across many chromosomes. These procedures resulted in a final sample size of 6,533 subjects in the TwinGene project, from which we randomly selected 2,000 subjects to form the basis for simulation; and a final sample size of 8,547 subjects (3,637 cases with schizophrenia and 4,820 controls) in the Swedish schizophrenia study. To obtain rare CNVs, we imposed a 0.01 frequency threshold in PLINK by removing CNVs with > 50% of its length spanning a region with > 85 CNVs in the Swedish schizophrenia CNV datasets or with > 65 CNVs in the TwinGene CNV dataset. We used the PLINK region-based method with a soft definition of intersection (i.e., 50% overlap) because it is a standard practice in data analysis projects and allows the evaluation of real-life scenarios. This filtering step resulted in PLINK format rare CNV files, where each file lists the base pair position (start and end) and copy number (or dosage coded as 0,1,3,4+) of individual CNV segments. From the PLINK format CNV file, we formed CNV regions (CNVR) by clustering individual segments using ≥1bp overlap. For each dataset, we then created the four required input matrixes as previously described in section “Statistical method”. For evaluating gene intersection effects in simulated data, we focused on 668 genes making proteins found in the neuronal postsynaptic density (PSD). Significant enrichment of rare CNVs in the PSD genes in schizophrenia cases has been reported [8, 9]. Finally, all CNVs in the analytic dataset from the Swedish schizophrenia study are restricted to >100kb because these can be detected with high confidence using the combination of GWAS arrays and PennCNV [8]. For evaluating gene intersection effects in schizophrenia data, we focused on nine gene sets, where significant enrichments of large rare CNVs in schizophrenia cases have been previously reported [3, 8, 10]. All gene sets were established *a priori* and independently of this work and included genes making RNAs that bind to FMRP (fragile X mental retardation protein, the product of *FMR1*) [3, 8, 10, 11], genes making proteins found in the PSD [9], genes implicated in mental retardation [12-15], expert-curated lists of synaptic genes [16], the “genes2cognition” database [17], genes spanned by *de novo* CNVs reported in Kirov et al [9].

**Reference:**

1. Beekman M, Heijmans BT, Martin NG, Whitfield JB, Pedersen NL, DeFaire U, et al. Two-locus linkage analysis applied to putative quantitative trait loci for lipoprotein(a) levels. Twin Res. 2003;6(4):322-4. doi: 10.1375/136905203322296692. PubMed PMID: 14511440.

2. Heijmans BT, Beekman M, Putter H, Lakenberg N, van der Wijk HJ, Whitfield JB, et al. Meta-analysis of four new genome scans for lipid parameters and analysis of positional candidates in positive linkage regions. Eur J Hum Genet. 2005;13(10):1143-53. doi: 10.1038/sj.ejhg.5201466. PubMed PMID: 16015283.

3. Ripke S, O'Dushlaine C, Chambert K, Moran JL, Kahler AK, Akterin S, et al. Genome-wide association analysis identifies 13 new risk loci for schizophrenia. Nat Genet. 2013;45(10):1150-9. doi: 10.1038/ng.2742. PubMed PMID: 23974872; PubMed Central PMCID: PMC3827979.

4. Lichtenstein P, Bjork C, Hultman CM, Scolnick EM, Sklar P, Sullivan PF. Recurrence risks for schizophrenia in a Swedish national cohort. Psychol Med. 2006;36:1417-26. PubMed PMID: 16863597.

5. Lichtenstein P, Sullivan P, Cnattingius S, Gatz M, Johansson S, Carlström C, et al. The Swedish Twin Registry in the Third Millennium – an update. Twin Res Hum Genet. 2006;9:875-82.

6. Pedersen NL, Lichtenstein P, Svedberg P. The Swedish Twin Registry in the Third Millenium. Twin Research. 2002;5:427-32.

7. Wang K, Li M, Hadley D, Liu R, Glessner J, Grant SF, et al. PennCNV: an integrated hidden Markov model designed for high-resolution copy number variation detection in whole-genome SNP genotyping data. Genome Res. 2007;17(11):1665-74. PubMed PMID: 17921354.

8. Szatkiewicz JP, O'Dushlaine C, Chen G, Chambert K, Moran JL, Neale BM, et al. Copy number variation in schizophrenia in Sweden. Mol Psychiatry. 2014. doi: 10.1038/mp.2014.40. PubMed PMID: 24776740.

9. Kirov G, Pocklington AJ, Holmans P, Ivanov D, Ikeda M, Ruderfer D, et al. De novo CNV analysis implicates specific abnormalities of postsynaptic signalling complexes in the pathogenesis of schizophrenia. Molecular psychiatry. 2011. Epub 2011/11/16. doi: 10.1038/mp.2011.154. PubMed PMID: 22083728.

10. Purcell SM, Moran JL, Fromer M, Ruderfer D, Solovieff N, Roussos P, et al. A polygenic burden of rare disruptive mutations in schizophrenia. Nature. 2014;506(7487):185-90. doi: 10.1038/nature12975. PubMed PMID: 24463508.

11. Darnell JC, Jensen KB, Jin P, Brown V, Warren ST, Darnell RB. Fragile X mental retardation protein targets G quartet mRNAs important for neuronal function. Cell. 2001;107(4):489-99. PubMed PMID: 11719189.

12. Chiurazzi P, Schwartz CE, Gecz J, Neri G. XLMR genes: update 2007. European journal of human genetics : EJHG. 2008;16(4):422-34. Epub 2008/01/17. doi: 10.1038/sj.ejhg.5201994. PubMed PMID: 18197188.

13. Inlow JK, Restifo LL. Molecular and comparative genetics of mental retardation. Genetics. 2004;166(2):835-81. Epub 2004/03/17. PubMed PMID: 15020472; PubMed Central PMCID: PMC1470723.

14. McKusick VA. Mendelian Inheritance in Man and its online version, OMIM. Am J Hum Genet. 2007;80(4):588-604. PubMed PMID: 17357067.

15. Najmabadi H, Hu H, Garshasbi M, Zemojtel T, Abedini SS, Chen W, et al. Deep sequencing reveals 50 novel genes for recessive cognitive disorders. Nature. 2011;478(7367):57-63. Epub 2011/09/23. doi: 10.1038/nature10423. PubMed PMID: 21937992.

16. Ruano D, Abecasis GR, Glaser B, Lips ES, Cornelisse LN, de Jong AP, et al. Functional gene group analysis reveals a role of synaptic heterotrimeric G proteins in cognitive ability. American journal of human genetics. 2010;86(2):113-25. Epub 2010/01/12. doi: 10.1016/j.ajhg.2009.12.006. PubMed PMID: 20060087; PubMed Central PMCID: PMC2820181.

17. Croning MD, Marshall MC, McLaren P, Armstrong JD, Grant SG. G2Cdb: the Genes to Cognition database. Nucleic acids research. 2009;37(Database issue):D846-51. Epub 2008/11/06. doi: 10.1093/nar/gkn700. PubMed PMID: 18984621; PubMed Central PMCID: PMC2686544.
